# Supplementary figures and images for: A toolbox for ablating excitatory and inhibitory synapses
Source: eLife. 2025 Apr 29;13:RP103757. doi: 10.7554/eLife.103757 (PMC12040315; doi:10.7554/eLife.103757)

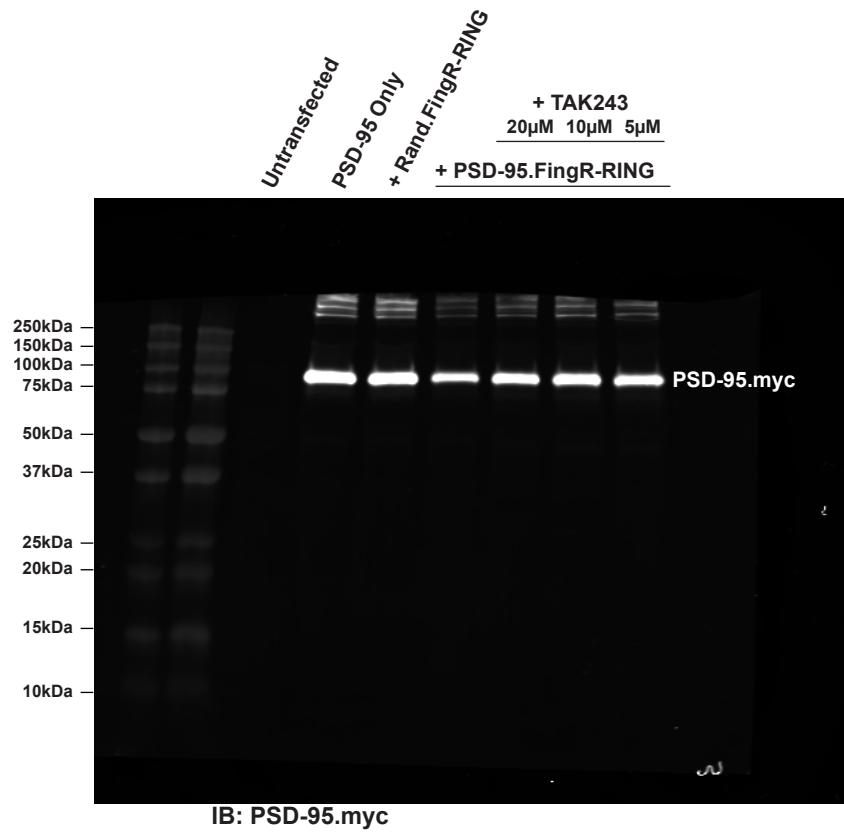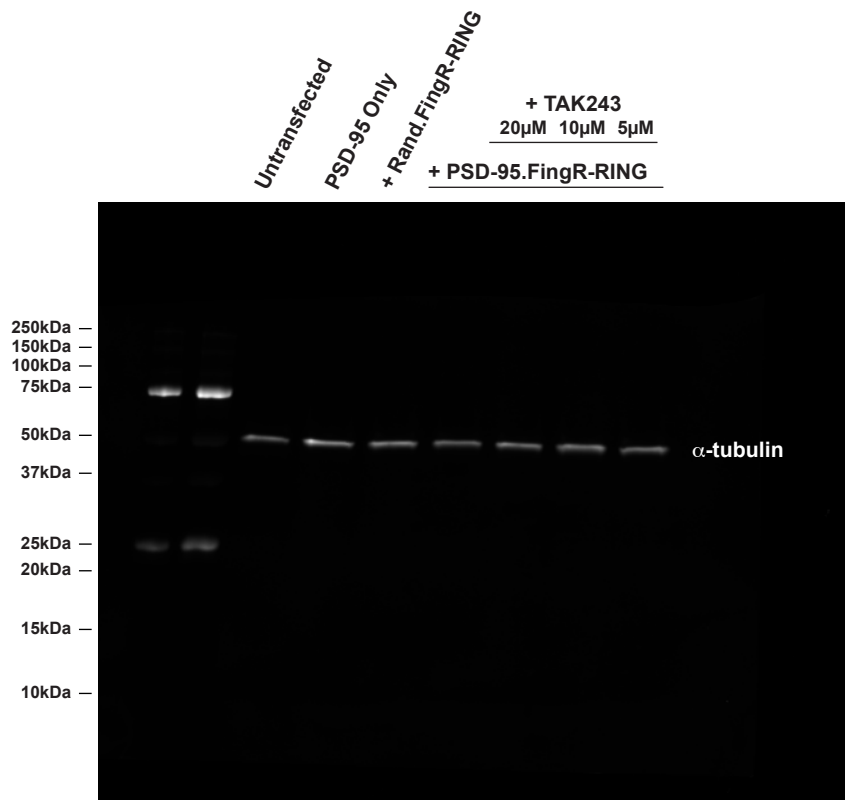

Supplement: Figure 1—source data 1. — Annotated western blots for PSD-95 and tubulin. [file elife-103757-fig1-data1.pdf]

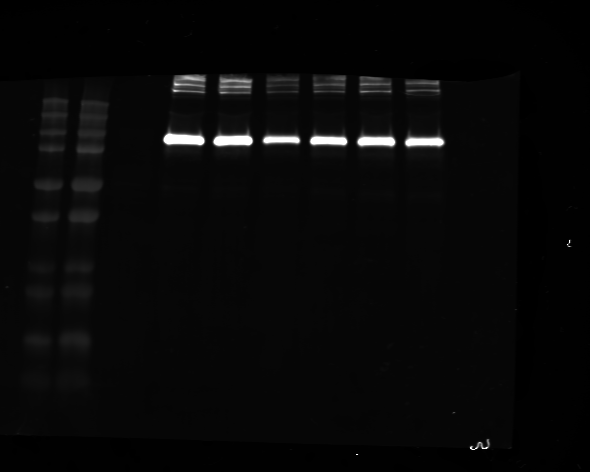

Supplement: Figure 1—source data 2. — Original western blots for PSD-95 and tubulin. [file elife-103757-fig1-data2.zip › PSD95 fig 1a RGB.tif]

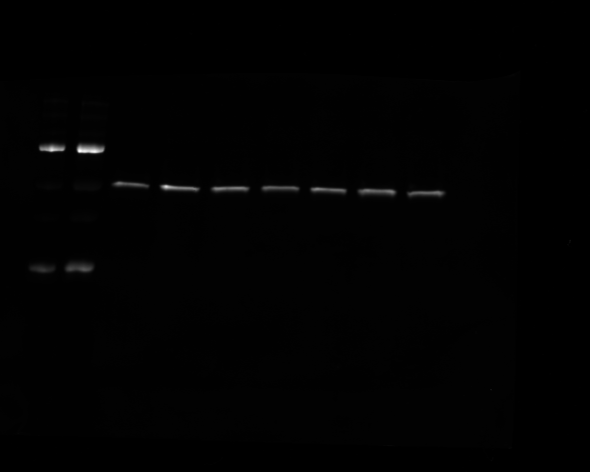

Supplement: Figure 1—source data 2. — Original western blots for PSD-95 and tubulin. [file elife-103757-fig1-data2.zip › tubulin fig 1a RGB.tif]

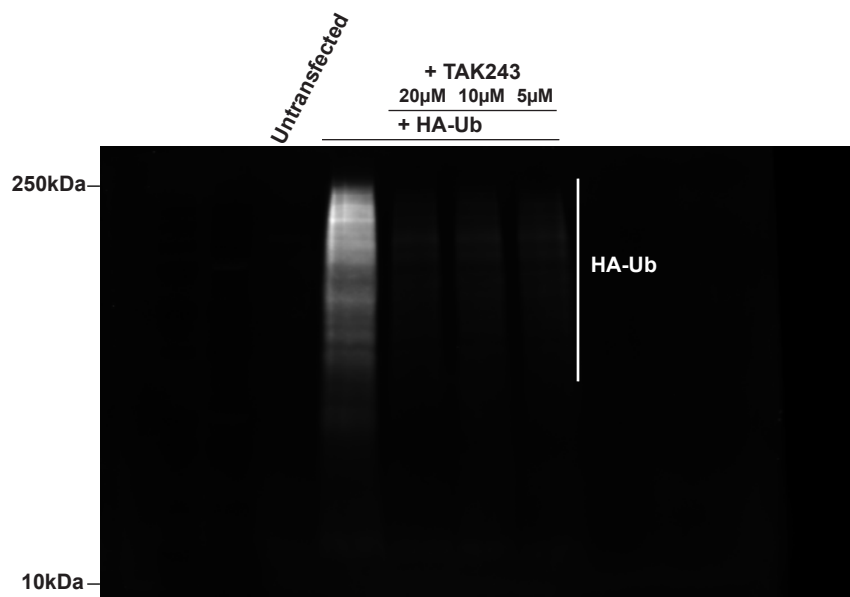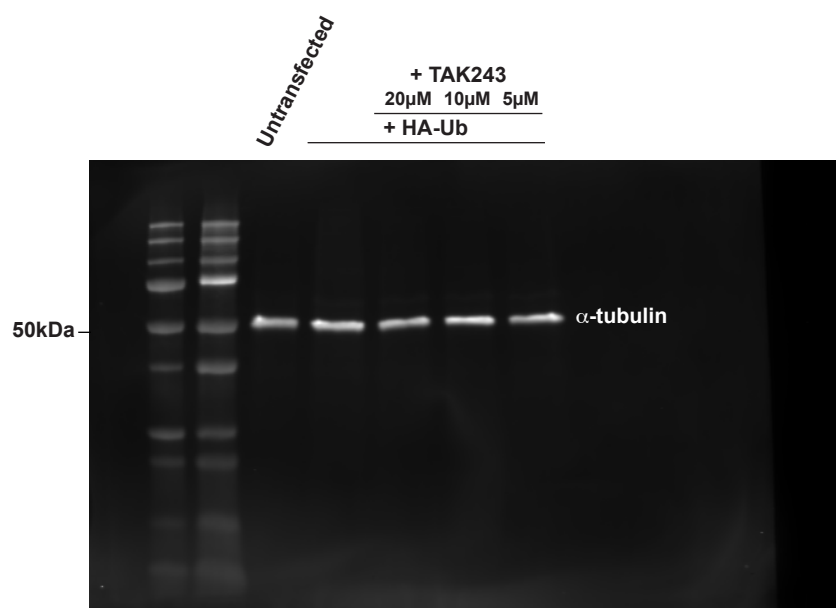

Supplement: Figure 1—figure supplement 1—source data 1. [file elife-103757-fig1-figsupp1-data1.pdf]

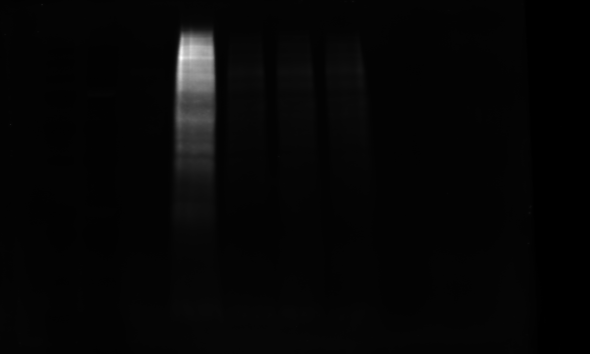

Supplement: Figure 1—figure supplement 1—source data 2. [file elife-103757-fig1-figsupp1-data2.zip › figure 1 supplement HA ub RGB.tif]

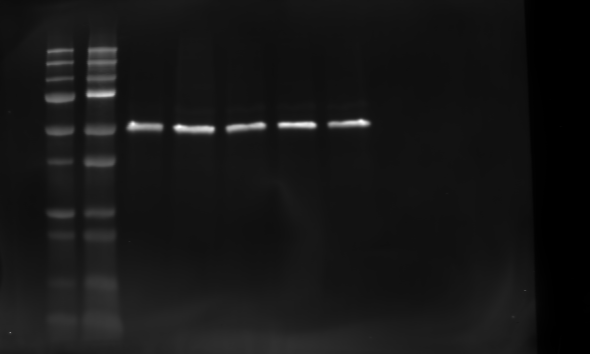

Supplement: Figure 1—figure supplement 1—source data 2. [file elife-103757-fig1-figsupp1-data2.zip › figure 1 supplement tubulin RGB.tif]

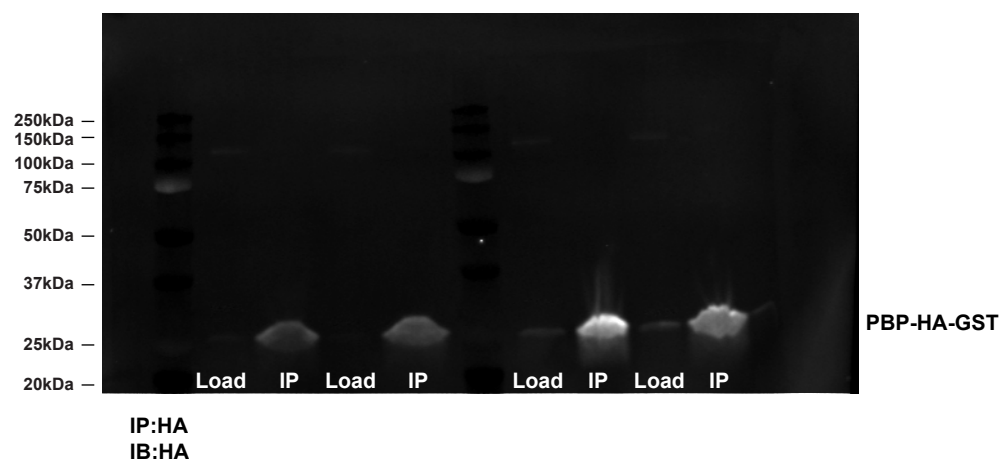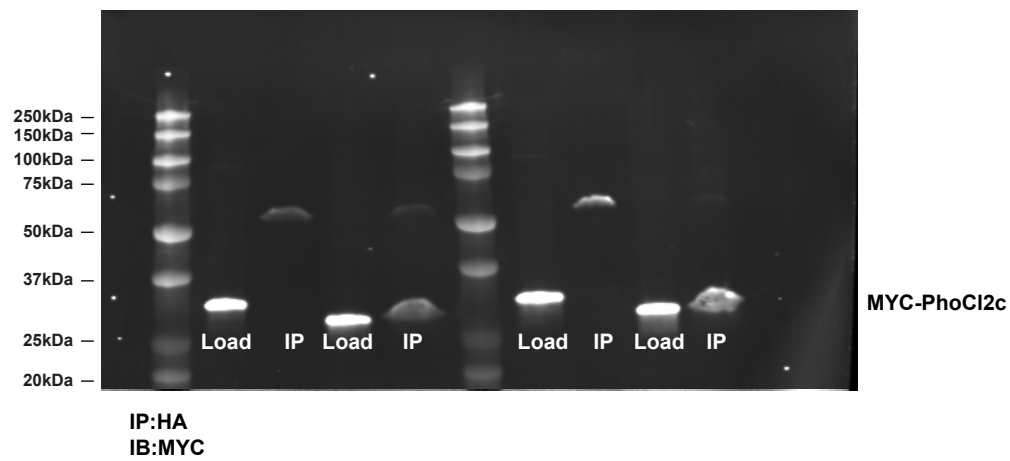

Supplement: Figure 4—source data 1. — Annotated western blots for PBP-HA-GST and Myc-PhoCl2c. [file elife-103757-fig4-data1.pdf]

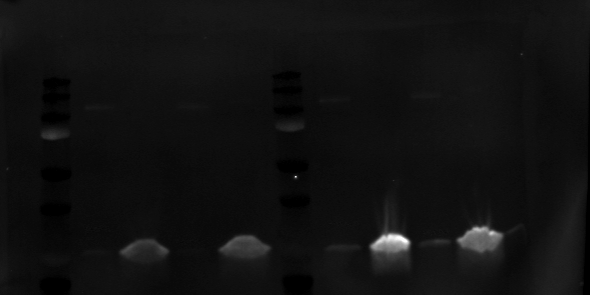

Supplement: Figure 4—source data 2. — Original western blots for PBP-HA-GST and Myc-PhoCl2c. [file elife-103757-fig4-data2.zip › Figure 4 source file 2 anti-HA immunoblot.TIF]

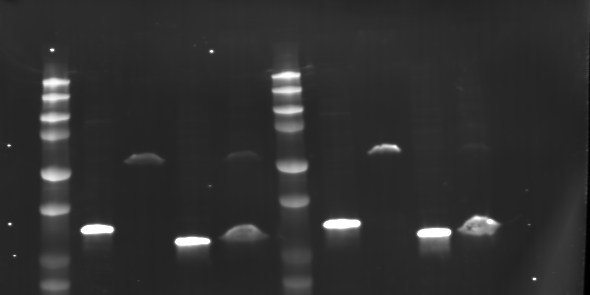

Supplement: Figure 4—source data 2. — Original western blots for PBP-HA-GST and Myc-PhoCl2c. [file elife-103757-fig4-data2.zip › Figure 4 Source file 3 anti-MYC immunoblot.TIF]
